# Supplementary material for: The Feasibility of a Text-Messaging Intervention Promoting Physical Activity in Shift Workers: A Process Evaluation
Source: Int J Environ Res Public Health. 2023 Feb 13;20(4):3260. doi: 10.3390/ijerph20043260 (PMC9962854; doi:10.3390/ijerph20043260)
Supplement: Supplementary file 1 [file ijerph-20-03260-s001.zip › ijerph-2188845-supplementary/Suplementary file_/Sup File 2-Exit online survey.pdf]

The feasibility of a text-messaging intervention promoting physical activity in shift workers: a process evaluation

<u>Exit online survey</u>

The following survey items addressing the implementation, context and mechanisms of action will be used:

**Process evaluation component**	**Survey items**
Reach	Why did you take part in th program
Adoption	Overall, how beneficial do you think the program has been to you so far?<br>How would you rate the text messages?<br>How would you rate the *SEMA*[3] app?<br>How would you rate the discussions with the researcher on the program?<br><br>(Answer with 1=no benefit, 5= neutral &; 10= extremely beneficial)
Implementation	Did the program meet your expectations?<br>Did you experience any barriers to participation in the program?<br>If you answered yes to the last question, please give details about any barriers:<br>Do you have any other comments about the health promotion program? (Maybe positive or negative factors).
Maintenance	I will continue to be physically active even after the program (answer: yes, uncertain, no)<br>Would you recommend this type of program to other shift workers?
