# Supplementary material for: The Feasibility of a Text-Messaging Intervention Promoting Physical Activity in Shift Workers: A Process Evaluation
Source: Int J Environ Res Public Health. 2023 Feb 13;20(4):3260. doi: 10.3390/ijerph20043260 (PMC9962854; doi:10.3390/ijerph20043260)
Supplement: Supplementary file 1 [file ijerph-20-03260-s001.zip › ijerph-2188845-supplementary/Suplementary file_/Sup File 3-Interview guide guide questions.pdf]

The feasibility of a text-messaging intervention promoting physical activity in shift workers: a process evaluation

Interview guide questions

1. Why did you participate in the program?
2. How has this intervention influenced your physical activity or exercise habits?
3. Which factors influenced your physical activity habits while on the intervention?
4. What were the most helpful and the least helpful components of the intervention?
5. How did you like the physical activity information provided?
6. What was your experiences about the text messages?
   -Follow up; what are some of the things you find most valuable/useful for you? And what things have you found not find valuable/useful?
7. Can you tell me about your experiences of using the SEMA app?
8. How has the program influenced your sleep habits?
9. How has the program influenced your sitting habits?
10. On the scale of 1-10, where 1 is not at all, and 10 is extremely confident. How competent do you feel to carry on being physically active after the programme has ended?
11. Have you used the *Mi* band you received as an incentive after the program? And has it played a role in your PA, sitting and sleeping?
12. Would you recommend this type of intervention for other people doing shift work who want to increase the amount of physical activity they do – why/why not?
